# Cognitive Outcomes in Young Adults with Primary Arterial Hypertension: The Role of Cardiovascular Risk Factors and Hypertension-Mediated Organ Damage

**DOI:** 10.3390/medicina60081353

**Published:** 2024-08-20

**Authors:** Kristijonas Puteikis, Karolis Ažukaitis, Danguolė Dadurkevičienė, Dovilė Mikučionytė, Kazys Simanauskas, Vaida Šileikienė, Rytis Masiliūnas, Augustina Jankauskienė, Rūta Mameniškienė

**Affiliations:** 1 Faculty of Medicine, Vilnius University, 03101 Vilnius, Lithuania; 2 Vilnius University Hospital Santaros Klinikos, 08406 Vilnius, Lithuania

**Keywords:** attention, cardiovascular risk, cognition, hypertension-mediated organ damage, memory, neuropsychology

## Abstract

*Background and Objectives:* We aimed to explore the association between cognitive performance and markers of hypertension-mediated organ damage (HMOD) in young adults with hypertension. *Materials and Methods:* A group of adults aged 16 to 45 years with primary arterial hypertension completed a battery of paper-pencil as well as computer-based neuropsychological tests across all major cognitive domains. They also underwent office and ambulatory 24 h blood pressure, intima-media thickness measurements, heart ultrasound, and laboratory analysis of their lipid profile, blood uric acid concentration, and urine albumin-creatinine ratio. Associations between cognitive test results and markers of HMOD were explored through correlation analysis and age-, sex-, and body mass index-adjusted linear regression modeling. *Results:* Seventy-six individuals (62, 81.6% male) aged 36.5 years (interquartile range 18.4 to 42.0 years) were enrolled. After adjusting for age, sex, and body mass index in linear regression models, worse categorical fluency was associated with higher left ventricular mass (β_st_ = −0.264, *p* = 0.043) and worse performance in a task of sustained attention—with higher left ventricular mass index (β_st_ = −0. 304, *p* = 0.016). Worse phonemic fluency was related to higher pulse pressure (β_st_ = −0.241, *p* = 0.049) in the respective model. Better strategy use in the task of spatial working memory was linked to higher daytime mean diastolic blood pressure (β_st_ = −0.343 *p* = 0.017). *Conclusions:* Performance among young adults with hypertension across selected cognitive domains was inversely associated with pulse pressure, markers of left ventricular damage, and directly associated with daytime diastolic blood pressure. Our study suggests that the previously reported relationship between cognitive and cardiovascular markers in hypertension exists earlier than in middle or late adulthood.

## 1. Introduction

Hypertension is a widespread and burdensome condition that greatly increases cardiovascular morbidity and mortality, making it one of the global health priorities [1]. While the more serious sequelae of poorly managed hypertension are numerous and include potentially lethal events, such as heart attacks or stroke, the risk of such outcomes can be mitigated by addressing the impact of hypertension on the cardiovascular and renal systems through early detection of subclinical hypertension-mediated organ damage (HMOD). The 2023 European Society of Hypertension (ESH) Guidelines for the management of arterial hypertension suggest specific clinical criteria for defining hypertension-mediated damage to the heart, peripheral arteries, eyes, and kidneys [2].

While the brain is acknowledged as another important target organ that is susceptible to damage induced by hypertension, no robust biomarkers have yet been suggested to define the presence of such injury. The Mini Mental State Examination and the Montreal Cognitive Assessment are proposed as routine screening methods in this context, but they are not specific to HMOD and, due to their low capability to detect subtle alterations, are applicable only in older populations [2,3]. Despite the known societal and economic importance of preserving brain health throughout the lifespan [4,5], cognitive outcomes in relation to hypertension have been understudied and have not undergone rigorous harmonization procedures characteristic of laboratory or imaging methods used to assess HMOD in the cardiovascular or renal systems. This is especially true in younger populations that are not yet at risk of dementia but have nevertheless been posited to experience negative effects of early hypertension on cognitive health in middle adulthood—the most robust evidence supporting this notion stems from the Coronary Artery Risk Development in Young Adults (CARDIA) Study [6,7] as well as the Young Finns Study [8].

Even considering the studies that suggest negative alterations in cognitive functioning among young adults with hypertension, the mechanisms of the observed changes have not been fully elucidated. It is well-known that primary hypertension is a condition extending well beyond the isolated elevation of blood pressure (BP). The accumulation of different cardiovascular risk factors (CVRF) may exert independent effects on neurocognitive outcomes. In addition, prior studies have shown an overlap of HMOD patterns in patients with hypertension, including structural and functional measures of cardiac and arterial injuries. Whether cognitive outcomes are associated with well-known HMOD markers and, thus, may share common pathways has not been extensively studied, particularly in younger adults with hypertension, where multimorbidity is less pronounced. Using a neuropsychological battery combining paper-pencil and computerized testing, we attempted to address the existing gap in the literature by exploring the association between CVRF, markers of HMOD, and cognitive performance in a group of young adults with hypertension.

## 2. Materials and Methods

### 2.1. Study Setting and Participants

We performed a cross-sectional study at Vilnius University Hospital Santaros Klinikos between 1 June 2021, and 30 June 2023. A convenience sample of young adults with primary arterial hypertension was formed from patients who had arrived for a routine medical consultation at the Centre for Family Medicine or the pediatric cardiology outpatient clinic.

Participant inclusion criteria were: (i) young adults (age 16–45 years), (ii) Lithuanian speakers, (iii) primary arterial hypertension (treated or non-treated) as defined by the European Society of Cardiology (ESC) and the European Society of Hypertension (ESH) guidelines of 2018 and 2016 (for those <18 years of age) [9,10]. Exclusion criteria were: (i) significant deficits (sensory or motor) preventing completion of cognitive evaluation, (ii) cardiovascular comorbidities besides primary arterial hypertension (e.g., congenital heart disease related to hypoxia or hypoperfusion, heart rhythm or conduction disorders with hemodynamic significance), (iii) diabetes mellitus, and (iv) glomerular filtration rate < 60 mL/min/1.73 m^2^ upon enrollment.

### 2.2. Cognitive Evaluation

The neuropsychological test battery consisted of paper-pencil and computerized (Cambridge Neuropsychological Test Automated Battery (CANTAB) by Cambridge Cognition, Ltd., Cambridge, UK) tests.

The testing battery included the following:

1. Categorical verbal fluency (count variable of different animals listed in 60 s).
2. Phonemic verbal fluency (number of nouns starting with “P” listed in 60 s).
3. Verbal-logical story recall (VLS, verbal memory). Participants listened to a short verbal story and were asked to recall it immediately, after 30 min, and after 24 h. The story was scored on a 24-point scale for each item recited.
4. Intelligence quotient (IQ) measured by the Wechsler Abbreviated Scale of Intelligence-II.
5. Nonverbal CANTAB tests (described in detail in Supplementary Table S1):
  (1) Match to Sample Visual Search (MTS): attention and processing speed.
  (2) Paired Associates Learning (PAL): visual memory and new learning.
  (3) Reaction Time Task (RTI): motor and mental response speed, movement time, reaction time, response accuracy, and impulsivity.
  (4) Rapid Visual Information Processing (RVP): sustained attention.
  (5) Stockings of Cambridge (SOC): spatial planning, working memory.
  (6) Spatial Span (SSP): working memory.
  (7) Spatial Working Memory (SWM): working memory and strategy use.

All tasks were performed by a licensed clinical psychologist in a single session conducted before ambulatory blood pressure monitoring (ABPM) measurement, except for IQ testing and 24-h VLS testing, which were performed upon taking off the ABPM cuff the second day. CANTAB tests were performed in accordance with the guidelines supplied by Cambridge Cognition, Ltd. The combined time required for neuropsychological evaluation was around 2 h for each participant. Situational factors, such as stress and fatigue, were mitigated through patient education and the use of an optimized examination protocol.

### 2.3. Cardiovascular Evaluation

Participants were measured for weight and height and underwent office BP (oBP) and ABPM measurements using validated oscillometric devices (ABPM: Mobile-O-Graph monitor, IEM, Aachen, Germany (18+ years), OnTrak Intertek 315762 device, Spacelabs Healthcare, Snoqualmie, Washington, United States (16–17 years)) in accordance with ESC/ESH guidelines, including recommendations for measurement conditions and posture [9,10]. The mean systolic (SBP) and diastolic (DBP) office BP values were calculated as the mean of the second and third measurements. Echocardiography was performed by an experienced cardiologist for all patients (Vivid 7 Ultrasound System, GE Healthcare, Chicago, Illinois, United States (18+ years), EPIQ diagnostic ultrasound system USN20B0120, Philips, Amsterdam, the Netherlands (16–17 years)). The left ventricular mass index (LVMI) was calculated as LVM/height (g/m^2.7^) according to ESH guidelines [2]. Intima-media thickness (IMT) was measured for all patients on both sides of the common carotid artery (the values were then averaged to yield a single measure) according to the Mannheim protocol (MyLab™X7, ESAOTE, Genoa, Italy (18+ years), EPIQ diagnostic ultrasound system USN20B0120 automated edge detection, Philips, Amsterdam, the Netherlands (16–17 years)) [11]. Patients provided a fasting blood sample to test for creatinine, uric acid, total cholesterol, high-density lipoprotein (HDL), low-density lipoprotein (LDL), and triglyceride concentrations. A morning urine sample was collected to measure the urine albumin-creatinine ratio (uACR).

LVH was defined as LVMI ≥ 50 g/m^2.7^ and ≥47 g/m^2.7^ for males and females, respectively, while LV concentric remodeling was defined as RWT ≥ 0.43 [2]. Mean IMT values > 0.9 mm were used to define the presence of atherosclerosis. Urine ACR values > 30 mg/g (>3.39 mg/mmol) were used for albuminuria, according to the ESH guidelines [2]. BMI values equal to or higher than 25 or 30 g/m^2^ were used to classify patients as overweight and obese, respectively.

### 2.4. Statistical Analysis

The normality of continuous variables was assessed using the Kolmogorov-Smirnov test. Correlation coefficients (Spearman’s rho) were calculated to determine the association between the cognitive and cardiovascular measures. All cardiovascular variables having statistically significant correlations with a single cognitive measure (the dependent variable) were then included as independent variables in a stepwise regression model, alongside age, sex, and body mass index (BMI). Cardiovascular variable(s) found to be statistically significant in the stepwise model were then entered into a simple regression model adjusted for age, sex, and BMI.

All statistical tests were two-tailed, and the level of significance was set at *p* < 0.05. All analyses were performed using IBM SPSS v26. A sample size of n = 77 was sought to yield a power of 0.8, α = 0.05 for a linear multiple regression model with three predictors and an effect size of f^2^ = 0.15 (G*Power 3.1.9.7).

### 2.5. Ethics

The study was approved by the Vilnius Regional Biomedical Research Ethics Committee (approval no. 2021/5-1348-821) and conducted in accordance with the principles of the World Medical Association Helsinki Declaration as well as national regulations. All participants (and parents or legal guardians, in the case of participants who were <18 years old) provided written informed consent upon enrollment.

## 3. Results

### 3.1. General Results

The study sample that completed the neuropsychological assessment consisted of 76 individuals (62, 81.6% male) aged 36.5 years (interquartile range 18.4 to 42 years). Forty-six (60.5%) of them had completed tertiary and 10 (13.2%) secondary education, while the remaining participants were either high school students or had lower levels of education. The results of neuropsychological testing are presented in Table 1.

Considering the participants’ cardiovascular profile, 50 (65.8%) were undergoing pharmacological treatment for hypertension: 28 (56.0%) with antihypertensive medication from one, 18 (36.0%)—from two, 3 (6.0%)—from three and 1 (2.0%)—from five different classes of drugs. There were 31 (40.8%) and 28 (36.8%) overweight and obese participants, respectively. Five (6.6%) patients had LV hypertrophy (LVH), seventeen (22.4%) had LV concentric remodeling, six (7.9%)—carotid atherosclerosis, and one (1.3%)—albuminuria. Most patients had an LDL-cholesterol blood concentration of >1.8 mmol/L (18, 23.7%) or >2.6 mmol/L (53, 69.7%). Fourteen (18.4%) participants were smokers. Anthropometric, laboratory, and BP measurement data of the participants are presented in Table 2.

### 3.2. Correlation Data

A correlation heatmap between cognitive and cardiovascular measures is presented in Supplementary Figure S1.

### 3.3. Regression Analyses Based on Results from Verbal Fluency, Verbal Memory and Intelligence Tests

In a stepwise regression model, worse categorical verbal fluency was associated with higher LVM (β_st_ = −0.306, *p* = 0.009), while age, sex, BMI, LVMI, and RWT were excluded from the model (R^2^ = 0.081). In a simple model adjusted for age, sex, and BMI, LVM remained a statistically significant variable (β_st_ = −0.264, *p* = 0.043; R^2^ = 0.061).

**Figure 1:**
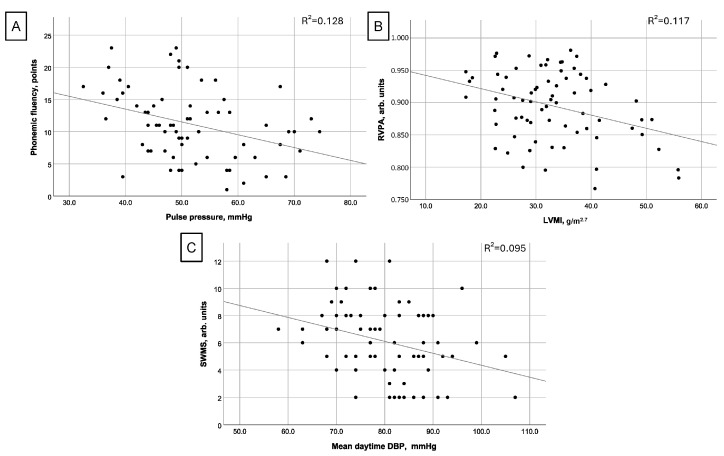
Scatterplots showing the relationship between the selected cardiovascular and cognitive variables, including a line approximating the best fit of the linear interaction. (correlations between pulse pressure and phonemic fluency (**A**), left ventricular mass index (LVMI) and results of the Rapid Visual Information Processing A’ prime test (RVPA, higher is better) that measures sustained attention (**B**), mean daytime diastolic blood pressure (DBP) and Spatial Working Memory test (SWMS, lower is better) results measuring working memory and strategy use (**C**)).

In a stepwise model (R^2^ = 0.192), worse phonemic fluency was associated with higher pulse pressure (PP, β_st_ = −0.362, *p* = 0.003) and uric acid concentration (β_st_ = −0.276, *p* = 0.020). Age (β_st_ = 0.277, *p* = 0.039) and PP (β_st_ = −0.241, *p* = 0.049, Figure 1A) remained significant variables in a simple regression model adjusted for age, sex, and BMI (R^2^ = 0.192).

While the 30 min verbal story recall was related to nighttime mean arterial pressure (β_st_ = 0.262, *p* = 0.028, R^2^ = 0.055) in a stepwise model, this relationship was no longer present after adjustment.

Immediate story recall (β_st_ = 0.361, *p* = 0.002, R^2^ = 0.117), verbal intelligence (β_st_ = 0.328, *p* = 0.013, R^2^ = 0.092), and general intelligence were associated only with age (β_st_ = 0.429, *p* < 0.001, R^2^ = 0.172).

### 3.4. Regression Analyses Based on Results from Computerized Cognitive Assessment

Despite the detected correlations between tests of attention and processing speed (MTS), visual memory and new learning (PAL), and response speed (RTI) with IMT (Supplementary Figure S1), such relationships were no longer statistically significant (*p* > 0.05) after adjustment for sex, age, and BMI in regression modeling.

A measure of sustained attention (RVPA) was inversely correlated with LVMI in a stepwise model (β_st_ = −0.335, *p* = 0.008, R^2^ = 0.097, *p* = 0.008), Figure 1B. This result persisted in a simple regression adjusted for sex, age, and BMI (β_st_ = −0.304, *p* = 0.016, R^2^ = 0.089). Finally, a higher daytime mean DBP was related to better performance (higher strategy use) in a spatial working memory task (SWMS, β_st_ = −0.343 *p* = 0.017, R^2^ = 0.081), Figure 1C.

## 4. Discussion

The aim of the current study was to explore how cognitive performance is related to cardiovascular profile and HMOD markers among young adults with primary arterial hypertension. We found statistically significant inverse associations between two cognitive domains—categorical verbal fluency and sustained attention—and LV parameters. Previous research has already shown that individuals with LV dysfunction tend to be at risk of white matter lesions, worse attention, executive performance, and global cognitive impairment, suggesting long-term hypertension exposure as a probable underlying factor for these findings [12,13,14]. On the other hand, recent reports revealed an independent relationship between brain imaging markers and LV parameters, thus questioning the role of hypertension alone as a causative factor of both cardiac and brain damage—a broader dysfunction of hormonal or stress responses (e.g., of the renin-aldosterone-angiotensin system) may be even more relevant [15,16]. It is essential to consider that the neuropsychological and imaging outcomes in earlier studies were evaluated in older populations, most often aged 65 years or more. While this helps to establish the potential relationship between left ventricle parameters and the risk of mild cognitive impairment and dementia later in life [17], it remains possible that the link between markers of subtle heart and brain damage could be detected even in young adults.

Evidence from the Bogalusa Heart Study revealed that, in individuals aged 48.4 ± 5.1 years, a fifth of the relationship between systolic blood pressure burden and cognition can be explained by a higher LVMI [18]. The authors suggested that LVMI may, therefore, be a central factor mediating the negative influence of hypertension on cognitive outcomes later in life. The notion of an independent association between cognitive functioning and cardiac damage is further supported by studies with longitudinal follow-up in middle-aged individuals [13,19,20]. Our findings extend previous research by providing evidence of an inverse association between categorical verbal fluency, sustained attention, and LV mass parameters in very young adults with hypertension. In accordance with the studies mentioned above, this relationship appears to be independent of blood pressure or other cardiovascular risk factors. However, LVM or LVMI explained only 6.7–8.9% of the variance in the results of the cognitive tests used. Therefore, this relationship, despite being statistically significant, can be expected to reflect only a part of the complex factors underlying variability in the two cognitive domains.

We also identified an association between low phonemic fluency and high PP, with PP accounting for 19.2% of the variance in this cognitive task. Similar to cardiac damage estimated through LV function, higher PP has been reported to be related to worsening in a broad spectrum of cognitive domains after a follow-up of middle-aged adults of several years [21]. In older individuals, higher PP is also associated with worse cognitive functioning (including mild cognitive impairment) [22], the level of Alzheimer’s disease biomarkers [23], white matter lesions [24], and reduced white matter neuronal fiber integrity [25]. The pathways of PP-associated damage to nervous tissue are thought to include disruption in cerebral blood flow autoregulation, resulting in microhemorrhages as well as endothelial dysfunction, oxidative stress, and inflammation [26,27,28]. While the negative influence of high PP has been emphasized in mid- and later life, our results suggest that it may be related to worse verbal fluency even in younger adults.

Another finding of our study was that high strategy use in a task, which is centered on finding hidden tokens, correlated with higher mean daytime DBP. As both direct [29,30] and inverse associations [31,32] between DBP and brain health have been identified in the literature, the role of DBP in neurocognition is now considered to be highly dependent on contextual factors, such as age, sex, and PP [29]. For example, better cognitive outcomes may be associated with higher DBP only if SBP is not elevated, and thus PP is low [32]. While it remains difficult to interpret why daytime DBP could be higher in those displaying better strategy use (possible explanations could include that higher DBP reflects lower arterial stiffness or better perfusion to eloquent brain regions), it should be noted that no other BP measures were statistically significant predictors of the tested cognitive outcomes in the regression models.

The results presented should be interpreted in light of the various limitations of our study. First, because of the time-consuming and extensive neuropsychological battery used, the sample size was small, providing limited statistical power to detect subtle associations between cognitive and cardiovascular variables or to create robust regression models containing more than several independent variables. It, therefore, remains unknown whether there are any additional associations between markers of HMOD or cardiovascular risk and cognitive functioning that remained undetected in the current study. On the other hand, the exploratory nature of the study and the inclusion of a comprehensive neuropsychological battery inflated the risk of Type I error across the spectrum of our analyses. The fact that most individuals included in the study were already treated for hypertension should also be considered, as this factor may lead to a blunted effect size when exploring the association between cognitive and BP variables. While the sample was predominantly male, this is most likely representative of population trends, with young men being significantly more affected by primary hypertension [33]. A more diverse patient group, from the perspective of ethnicity and socioeconomic background, is necessary to increase the generalizability of our findings. Furthermore, the characterization of our study sample was not extensive enough to account for societal, psychological, lifestyle, or environmental confounders that may have important associations with cognitive performance in early adulthood. We believe this notion was also reflected by the rather small R-squared values in the presented regression models, indicating that a more comprehensive evaluation of cognitive profiles should be performed by accounting for a broader physiological, demographic, and socioeconomic context. We also did not investigate the association between cognitive function and HMOD in other organ systems (beyond renal and cardiovascular dysfunction) that are also affected by arterial hypertension. For instance, retinal changes have been shown to be among the earliest markers of hypertensive damage to the microvasculature [34] and may, therefore, have a more evident correlation with cognitive outcomes among youth.

## 5. Conclusions

Our study extends previous research in middle-to-late adulthood by showing that certain cognitive domains, such as verbal fluency, sustained attention, and strategy use, are associated with left ventricle parameters, pulse pressure, and diastolic blood pressure, even in very young adults with hypertension. Further investigation of how these relationships are mediated could help guide clinical decisions aimed at brain health preservation and promotion several decades before the onset of mild cognitive impairment or dementia. Given the potential for a larger time window to address the impact of hypertension on cognitive health in clinical practice, we believe that our results should be replicated in larger young adult studies focused on the association between left ventricular function, blood pressure characteristics, and cognitive performance. Moreover, future studies should consider the inclusion of a more diverse patient population as well as a longitudinal study design to provide generalizable data about the causal interaction between hypertension burden and cognitive functioning among young individuals. Finally, additional data on the pathophysiological interaction between hypertension-induced microvascular changes and brain function are needed from fundamental and translational studies.

## Figures and Tables

**Table 1:** Results of participant performance across different neuropsychological tests. For detailed descriptions of separate variables, please refer to the section “Cognitive evaluation” and Supplementary Table S1.

Cognitive Test	N	Mean (SD) or Median (IQR)
Paper-pencil assessment		
Categorical verbal fluency, points ^h^	76	22.3 ± 7.0
Phonemic verbal fluency, points ^h^	76	11.2 ± 5.4
VLS immediate recall, points ^h^	76	15.5 (13.1–17.4)
VLS 30 min recall, points ^h^	76	13.2 ± 3.9
VLS 24 h recall, points ^h^	72	12.8 ± 4.1
Verbal IQ, points ^h^	72	112.5 (100.3–120.0)
Nonverbal IQ, points ^h^	74	112 (99.8–118.3)
IQ, points ^h^	68	115 (105–123)
Computerized assessment		
*Match to Sample Visual Search*		
MTSPS82, ms ^c^	76	2131.6 (1738.5–2583.3)
MTSRCAMD, ms ^l^	76	2174.8 (1892.8–2623.4)
*Paired Associates Learning*		
PALFAMS28, points ^h^	76	14.2 ± 3.8
PALTEA28, points ^l^	76	9 (3.0–14.8)
*Reaction Time Task*		
RTIFMDMT, ms ^l^	76	282.8 ± 66.1
RTIFMDRT, ms ^l^	76	357.5 (338.0–386.9)
RTISMDMT, ms ^l^	76	242.5 (199.0–290.9)
RTISMDRT, ms ^l^	76	321.3 (305.6–344.4)
*Rapid Visual Information* *Processing*		
RVPA, arb. unit ^h^	76	0.9 ± 0.1
RVPMDL, ms ^l^	76	441.3 (409.0–506.5)
RVPPFA, arb.unit ^l^	76	0 (0–0)
*Stockings of Cambridge*		
SOCITMD5, ms ^l^	76	10,283.3 (7170.0–15,765.8)
SOCMNM5, points ^l^	76	5.6 (5.0–6.5)
SOCPSMMT, points ^h^	76	9.5 (9.0–11)
SOCSTMD5, ms ^l^	76	0 (0–410.6)
*Spatial Span*		
SSPFSL, points ^h^	76	7 (6–8)
*Spatial Working Memory*		
SWMBE4, points ^l^	76	0 (0–0)
SWMBE468, points ^l^	76	3 (0–11)
SWMBE6, points ^l^	76	0 (0–2)
SWMBE8, points ^l^	76	1 (0–9)
SWMS, arb. unit ^l^	76	6 (4–8)

c—complex interpretation, l—lower is better, h—higher is better, IQ—intelligence quotient, MTS—Match to Sample Visual Search, PAL—Paired Associates Learning, RTI—Reaction Time Task, RVP—Rapid Visual Information Processing, SOC—Stockings of Cambridge, SSP—Spatial Span, SWM—Spatial Working Memory, VLS—verbal-logical story task.

**Table 2:** Anthropometric, laboratory and blood pressure measurement data of the study participants.

Characteristic	Mean (SD) or Median (IQR)
Age, years	36.5 (18.4–42.0)
BMI, kg/m^2^	28.8 ± 5.0
SBP, mmHg	134 ± 16
DBP, mmHg	80 (75–89)
PP, mmHg	50 (45–58)
Creatinine, µmol/L	79.3 ± 11.4
Uric acid, µmol/L	384.2 ± 78.9
uACR, mg/mmol	0.5 (0.3–0.8)
Cholesterol, mmol/l	5.2 ± 1.2
LDL, mmol/L	3.3 ± 1.0
HDL, mmol/L	1.2 ± 0.3
Triglycerides, mmol/L	1.4 (0.9–2.0)
LVM, g	162.6 ± 43.4
LVMI, g/m^2.7^	33.1 ± 8.9
RWT	0.38 ± 0.07
EF, %	67 ± 9
24-h SBP, mmHg	128 ± 10
24-h DBP, mmHg	78 ± 9
24-h MAP, mmHg	99 ± 10
Daytime SBP, mmHg	131 ± 10
Daytime DBP, mmHg	80 ± 10
Daytime MAP, mmHg	102 ± 10
Nighttime SBP, mmHg	117 ± 10
Nighttime DBP, mmHg	68 (62.8–72.0)
Nighttime MAP, mmHg	90 ± 9
SBP dipping, %	10.4 ± 5.6
DBP dipping, %	14.5 ± 6.0
IMT (left), mm	0.58 (0.48–0.69)
IMT (right), mm	0.59 ± 0.14
IMT (median), mm	0.60 ± 0.14

BMI—body mass index, DBP—diastolic blood pressure, EF—ejection fraction, HDL—high-density lipoprotein cholesterol, IMT—intima-media thickness, LDL—low-density lipoprotein cholesterol, LVM—left ventricular mass, LVMI—left ventricular mass index, MAP—mean arterial pressure, PP—pulse pressure, RWT—relative wall thickness, SBP—systolic blood pressure, uACR—urine albumin-creatinine ratio.

## Data Availability

Pseudonymized data supporting the findings will be made available upon reasonable request, supported by a data analysis plan and approval by the Vilnius Regional Biomedical Research Ethics Committee.

## Supplementary Materials

The following supporting information can be downloaded at https://www.mdpi.com/article/10.3390/medicina60081353/s1: Figure S1. A heatmap representing correlations (Spearman’s correlation coefficients) between age, cognitive test, and cardiovascular variables. Table S1: Description of computerized cognitive CANTAB (Cambridge Cognition, Ltd.) measures used in the current study.
