# Supplementary material for: Cognitive Outcomes in Young Adults with Primary Arterial Hypertension: The Role of Cardiovascular Risk Factors and Hypertension-Mediated Organ Damage
Source: Medicina (Kaunas). 2024 Aug 20;60(8):1353. doi: 10.3390/medicina60081353 (PMC11356388; doi:10.3390/medicina60081353)
Supplement: Supplementary file 1 [file medicina-60-01353-s001.zip › medicina-3146578-supplementary.pdf]

| | Age, years | BMI, kg/m2 | SBP, mmHg | DBP, mmHg | PP, mmHg | Creatinine, µmol/L | Uric acid, µmol/L | uACR, mg/mmol | Cholesterol, mmol/l | LDL, mmol/L | HDL, mmol/L | Triglycerides, mmol/L | LVM, g | LVMI, g/m^2.7 | RWT | EF, % | 24-h SBP, mmHg | 24-h DBP, mmHg | 24-h MAP, mmHg | Daytime SBP, mmHg | Daytime DBP, mmHg | Daytime MAP, mmHg | Nighttime SBP, mmHg | Nighttime DBP, mmHg | Nighttime MAP, mmHg | SBP dipping, % | DBP dipping, % | IMT (left), mm | IMT (right), mm | IMT (median), mm |
|---|---|---|---|---|---|---|---|---|---|---|---|---|---|---|---|---|---|---|---|---|---|---|---|---|---|---|---|---|---|---|
| Categorical verbal fluency, points | 0.189 | 0.039 | -0.201 | -0.054 | -0.125 | 0.042 | -0.199 | -0.050 | 0.144 | 0.088 | 0.016 | 0.035 | **-0.239** | **-0.243** | **-0.258** | 0.072 | -0.052 | 0.104 | 0.079 | -0.023 | 0.126 | 0.088 | -0.062 | 0.056 | 0.072 | 0.108 | 0.088 | 0.065 | 0.063 | 0.028 |
| Phonemic verbal fluency, points | **0.360** | -0.028 | **-0.285** | -0.006 | **-0.365** | 0.078 | **-0.298** | 0.083 | 0.207 | 0.134 | 0.027 | 0.188 | -0.216 | -0.168 | **-0.297** | -0.073 | -0.096 | **0.259** | 0.159 | -0.089 | **0.254** | 0.165 | -0.086 | **0.270** | 0.184 | 0.089 | 0.003 | 0.118 | 0.165 | 0.122 |
| VLS immediate recall, points | **0.313** | 0.112 | -0.122 | 0.049 | -0.135 | -0.041 | -0.046 | 0.034 | 0.160 | 0.065 | -0.229 | **0.234** | -0.028 | 0.002 | -0.050 | 0.016 | 0.058 | 0.152 | 0.170 | 0.047 | 0.110 | 0.161 | 0.116 | 0.186 | 0.229 | -0.011 | -0.142 | 0.165 | 0.097 | 0.133 |
| VLS 30 min recall, points | 0.207 | 0.137 | -0.163 | -0.019 | -0.122 | -0.020 | -0.006 | 0.047 | 0.080 | 0.004 | -0.155 | 0.162 | -0.025 | 0.036 | -0.109 | 0.149 | 0.065 | 0.179 | 0.179 | 0.031 | 0.139 | 0.163 | 0.175 | 0.206 | **0.271** | -0.094 | -0.125 | 0.088 | 0.034 | 0.054 |
| VLS 24 h recall, points | 0.196 | 0.081 | -0.190 | -0.102 | -0.062 | 0.062 | 0.019 | 0.070 | 0.071 | -0.002 | -0.193 | 0.155 | -0.059 | -0.019 | -0.077 | 0.185 | 0.061 | 0.093 | 0.109 | 0.028 | 0.060 | 0.102 | 0.220 | 0.134 | 0.238 | -0.118 | -0.112 | 0.017 | 0.046 | 0.032 |
| Verbal IQ, points | **0.384** | -0.092 | -0.121 | 0.027 | **-0.243** | -0.088 | -0.231 | 0.122 | **0.295** | 0.209 | 0.122 | 0.089 | **-0.253** | -0.212 | -0.185 | -0.032 | -0.051 | 0.215 | 0.175 | -0.030 | 0.212 | 0.151 | -0.017 | **0.312** | **0.249** | 0.035 | -0.165 | 0.139 | **0.295** | 0.220 |
| Nonverbal IQ, points | 0.125 | -0.165 | -0.106 | -0.005 | -0.149 | 0.068 | -0.183 | 0.164 | 0.138 | 0.019 | 0.194 | 0.084 | -0.073 | -0.092 | -0.080 | -0.061 | -0.091 | 0.175 | 0.107 | -0.097 | 0.147 | 0.084 | -0.036 | 0.233 | 0.179 | -0.049 | -0.146 | -0.028 | 0.036 | -0.016 |
| IQ, points | 0.224 | -0.160 | -0.091 | 0.019 | -0.190 | -0.003 | -0.180 | 0.196 | 0.192 | 0.133 | 0.150 | 0.092 | -0.103 | -0.087 | -0.050 | -0.075 | -0.045 | 0.223 | 0.142 | -0.044 | 0.201 | 0.114 | -0.012 | **0.313** | 0.213 | -0.005 | -0.189 | -0.063 | 0.101 | 0.002 |
| MTSPS82, ms | **0.270** | 0.065 | 0.075 | 0.121 | -0.080 | -0.010 | 0.008 | 0.044 | **0.236** | **0.248** | 0.118 | -0.003 | -0.159 | -0.112 | -0.048 | -0.067 | 0.116 | 0.039 | 0.138 | 0.138 | 0.082 | 0.128 | 0.113 | 0.052 | 0.132 | -0.007 | 0.042 | **0.305** | **0.319** | **0.368** |
| MTSRCAMD, ms | **0.360** | 0.146 | 0.170 | 0.102 | 0.172 | 0.084 | 0.108 | -0.234 | 0.197 | 0.186 | -0.039 | 0.110 | -0.011 | 0.007 | 0.087 | **-0.239** | 0.004 | -0.064 | 0.028 | -0.019 | -0.056 | 0.011 | 0.042 | 0.001 | 0.049 | -0.056 | -0.057 | **0.307** | **0.289** | **0.323** |
| PALFAMS28, points | **-0.288** | -0.251 | -0.011 | -0.074 | 0.078 | 0.001 | -0.079 | 0.212 | -0.174 | -0.208 | 0.143 | **-0.242** | 0.139 | 0.084 | -0.108 | 0.068 | 0.133 | 0.016 | 0.024 | 0.199 | 0.043 | 0.065 | 0.003 | -0.119 | -0.069 | 0.207 | **0.243** | **-0.297** | -0.218 | **-0.276** |
| PALTEA28, points | **0.231** | 0.216 | 0.049 | 0.040 | -0.011 | 0.014 | 0.105 | -0.259 | 0.127 | 0.190 | -0.171 | 0.166 | -0.141 | -0.093 | 0.109 | -0.099 | -0.076 | -0.052 | -0.034 | -0.126 | -0.062 | -0.071 | 0.040 | 0.049 | 0.039 | -0.164 | -0.175 | **0.278** | 0.201 | **0.260** |
| RTIFMDMT, ms | 0.216 | 0.152 | **0.240** | 0.169 | 0.120 | -0.096 | -0.109 | -0.116 | 0.028 | 0.003 | -0.141 | 0.057 | -0.015 | 0.067 | 0.144 | -0.127 | 0.048 | -0.061 | -0.032 | -0.009 | -0.096 | -0.053 | 0.160 | 0.001 | 0.052 | -0.193 | -0.123 | **0.292** | 0.220 | **0.288** |
| RTIFMDRT, ms | **0.333** | 0.082 | 0.174 | 0.091 | 0.145 | 0.032 | 0.093 | -0.022 | 0.052 | 0.023 | -0.065 | 0.020 | 0.054 | 0.076 | 0.025 | -0.033 | -0.010 | -0.075 | -0.006 | -0.028 | -0.093 | -0.030 | 0.069 | 0.045 | 0.088 | -0.069 | -0.145 | **0.357** | **0.399** | **0.414** |
| RTISMDMT, ms | 0.182 | 0.179 | 0.087 | 0.116 | -0.028 | -0.144 | -0.164 | -0.107 | 0.034 | -0.017 | -0.159 | 0.158 | -0.035 | 0.016 | 0.027 | -0.082 | 0.069 | 0.002 | 0.035 | -0.014 | -0.048 | 0.002 | 0.153 | 0.053 | 0.110 | -0.223 | -0.099 | 0.216 | 0.180 | 0.215 |
| RTISMDRT, ms | **0.290** | -0.073 | 0.077 | -0.022 | 0.160 | 0.049 | 0.026 | -0.088 | 0.135 | 0.122 | -0.026 | 0.022 | 0.121 | 0.089 | 0.039 | -0.004 | 0.013 | -0.025 | 0.057 | -0.028 | -0.050 | 0.014 | 0.064 | 0.077 | 0.126 | -0.086 | -0.114 | **0.352** | **0.419** | **0.416** |
| RVPA, arb. unit | 0.109 | -0.099 | **-0.290** | -0.121 | -0.220 | 0.149 | -0.147 | 0.060 | 0.111 | 0.012 | 0.042 | 0.085 | -0.192 | **-0.246** | -0.168 | -0.005 | -0.047 | 0.151 | 0.106 | -0.067 | 0.105 | 0.079 | 0.083 | 0.224 | 0.233 | -0.102 | -0.148 | 0.032 | **0.258** | 0.140 |
| RVPMDL, ms | 0.113 | 0.105 | **0.265** | 0.142 | 0.197 | -0.122 | 0.071 | 0.254 | -0.071 | -0.045 | 0.050 | -0.076 | 0.052 | 0.056 | -0.011 | -0.078 | 0.054 | -0.036 | 0.001 | 0.038 | -0.049 | -0.015 | 0.122 | 0.060 | 0.114 | -0.163 | -0.143 | 0.182 | -0.024 | 0.086 |
| RVPPFA, arb.unit | 0.045 | **0.239** | **0.329** | **0.331** | 0.101 | -0.175 | 0.044 | -0.042 | 0.084 | 0.064 | 0.021 | 0.008 | 0.076 | 0.214 | 0.133 | 0.168 | 0.080 | 0.083 | 0.098 | 0.094 | 0.114 | 0.115 | 0.002 | 0.003 | -0.001 | -0.028 | 0.095 | 0.138 | -0.091 | 0.049 |
| SOCITMD5, ms | -0.103 | 0.039 | -0.142 | -0.168 | 0.008 | 0.075 | 0.043 | 0.027 | -0.129 | -0.124 | 0.159 | -0.128 | -0.108 | -0.141 | -0.003 | 0.215 | 0.028 | -0.136 | -0.103 | -0.010 | -0.147 | -0.109 | 0.132 | -0.077 | 0.008 | -0.190 | -0.073 | -0.063 | -0.039 | -0.053 |
| SOCMNM5, points | -0.078 | -0.014 | -0.033 | -0.033 | -0.020 | -0.049 | 0.114 | -0.126 | -0.044 | 0.024 | -0.127 | 0.092 | 0.204 | 0.101 | **0.235** | 0.056 | -0.023 | -0.098 | -0.083 | 0.014 | -0.118 | -0.069 | -0.118 | -0.132 | -0.154 | 0.104 | 0.039 | 0.119 | -0.140 | 0.009 |
| SOCPSMMT, points | -0.104 | -0.088 | 0.091 | 0.000 | 0.116 | -0.012 | -0.038 | 0.180 | -0.009 | -0.047 | **0.268** | -0.167 | -0.146 | -0.104 | -0.123 | 0.170 | 0.070 | 0.064 | 0.048 | 0.042 | 0.079 | 0.033 | 0.235 | 0.114 | 0.166 | -0.205 | -0.097 | -0.157 | 0.177 | 0.002 |
| SOCSTMD5, ms | -0.080 | 0.013 | -0.004 | -0.080 | 0.062 | -0.093 | 0.103 | -0.170 | -0.005 | 0.060 | -0.037 | -0.064 | 0.143 | 0.021 | 0.217 | 0.073 | -0.044 | -0.147 | -0.097 | -0.016 | -0.138 | -0.085 | -0.097 | -0.150 | -0.153 | 0.028 | 0.024 | 0.022 | -0.129 | -0.043 |
| SSPFSL, points | -0.127 | -0.182 | -0.073 | -0.035 | -0.002 | 0.196 | 0.078 | -0.018 | 0.061 | 0.011 | 0.207 | -0.074 | 0.092 | -0.027 | -0.015 | 0.031 | 0.061 | 0.038 | 0.013 | 0.091 | 0.037 | 0.012 | -0.005 | -0.024 | -0.039 | 0.103 | 0.069 | -0.180 | -0.110 | -0.175 |
| SWMBE4, points | 0.102 | 0.123 | 0.033 | -0.028 | 0.126 | 0.023 | 0.094 | -0.267 | 0.076 | 0.123 | -0.189 | **0.243** | 0.155 | 0.157 | 0.123 | -0.158 | -0.104 | -0.139 | -0.136 | -0.063 | -0.123 | -0.147 | -0.156 | -0.133 | -0.166 | 0.129 | 0.048 | 0.157 | 0.127 | 0.164 |
| SWMBE468, points | 0.034 | 0.097 | -0.058 | -0.051 | 0.006 | -0.177 | 0.086 | 0.070 | 0.043 | 0.069 | -0.087 | 0.175 | 0.106 | 0.117 | 0.147 | -0.116 | -0.014 | -0.206 | -0.141 | -0.008 | -0.222 | -0.153 | 0.000 | -0.156 | -0.132 | -0.044 | -0.043 | 0.161 | -0.050 | 0.082 |
| SWMBE6, points | 0.066 | 0.213 | 0.021 | -0.034 | 0.021 | -0.118 | 0.085 | 0.142 | 0.009 | 0.065 | -0.221 | 0.141 | 0.119 | 0.092 | 0.151 | -0.143 | 0.130 | -0.087 | 0.014 | 0.139 | -0.080 | -0.016 | 0.171 | -0.005 | 0.074 | -0.081 | -0.076 | 0.127 | 0.101 | 0.153 |
| SWMBE8, points | -0.044 | 0.089 | -0.079 | -0.063 | -0.011 | -0.196 | 0.161 | 0.100 | 0.055 | 0.057 | -0.032 | 0.151 | 0.119 | 0.118 | 0.173 | -0.056 | -0.050 | -0.223 | -0.166 | -0.047 | **-0.245** | -0.171 | -0.035 | -0.208 | -0.185 | -0.052 | -0.014 | 0.103 | -0.131 | -0.006 |
| SWMS, arb. unit | -0.083 | 0.086 | 0.026 | -0.090 | 0.183 | -0.137 | 0.163 | -0.103 | -0.100 | -0.101 | -0.103 | 0.167 | 0.203 | 0.178 | **0.277** | -0.058 | 0.050 | **-0.276** | -0.198 | 0.021 | **-0.306** | -0.219 | 0.077 | **-0.266** | -0.185 | -0.129 | -0.065 | 0.083 | -0.092 | 0.018 |

**Supplementary Figure S1.** A heatmap representing correlations (Spearman's correlation coefficients) between age, cognitive test and cardiovascular variables (stronger positive correlation coefficients are coloured in increasingly intense green while stronger negative correlation coefficients are coloured increasingly intense red). Statistically significant correlations are shown in bold and underlined. BMI – body mass index, DBP – diastolic blood pressure, EF – ejection fraction, HDL – high-density lipoprotein cholesterol, IMT – intima-media thickness, LDL – low-density lipoprotein cholesterol, LVM – left ventricular mass, LVMI – left ventricular mass index, MAP – mean arterial pressure, PP – pulse pressure, RWT – relative wall thickness, SBP – systolic blood pressure, uACR – urine albumin-creatinine ratio, IQ – intelligence quotient, MTS – Match to Sample Visual Search, PAL – Paired Associates Learning, RTI – Reaction Time Task, RVP – Rapid Visual Information Processing, SOC – Stockings of Cambridge, SSP – Spatial Span, SWM – Spatial Working Memory, VLS – verbal-logical story task (for detailed descriptions of separate variables please refer to the section "Cognitive evaluation" and Appendix A).

**Supplementary Table S1**

Description of computerized cognitive CANTAB (Cambridge Cognition, Ltd.) measures used in the current study.

| |
|---|
| **Match to Sample Visual Search (MTS)** |
| **Domains:** attention and processing speed.<br>**Description:** One pattern is displayed in the middle of the screen. The participant must select a matching pattern appearing in other boxes on the screen as rapidly as possible.<br>**Variables:**<br>MTSPS82 – The difference in mean time between presentation of the response stimulus options and the subject selecting the correct box on their first attempt on the 8 pattern assessment trials compared to the 2 pattern assessment trials. Calculated across 8 pattern and 2 pattern assessed trials where the first attempt was correct.<br>MTSRCAMD – The median time between presentation of the response stimulus options and the subject selecting the correct box (across all assessed trials). |
| **Paired Associates Learning (PAL)** |
| **Domains:** visual memory and new learning.<br>**Description:** The participant selects boxes with a matching pattern based on previously shown information.<br>**Variables:**<br>PALFAMS28 – The number of times a subject chose the correct box on their first attempt when recalling the pattern locations. Calculated across assessed trials, omitting 12 box level.<br>PALTEA28 – The number of times the subject chose the incorrect box for a stimulus on assessment problems, plus an adjustment for the estimated number of errors they would have made on any problems, attempts and recalls they did not reach. This measure allows to compare performance on errors made across all subjects regardless of those who terminated early versus those completing the final stage of the task. |
| **Reaction Time Task (RTI)** |
| **Domains:** motor and mental response speed, movement time, reaction time, response accuracy and impulsivity.<br>**Description:** the participant holds a button at the bottom of the screen and has to react to flashing circles above as soon as possible (release the button and tap the flashing circle). |

| |
|---|
| **Variables:**<br>RTIFMDMT – The median time taken for a subject to release the response button and select the target stimulus after it flashed yellow on screen. Calculated across correct, assessed trials in which the stimulus could appear in any one of five locations. Measured in milliseconds.<br>RTIFMDRT – The median duration it took for a subject to release the response button after the presentation of a target stimulus. Calculated across correct, assessed trials in which the stimulus could appear in any one of five locations. Measured in milliseconds.<br>RTISMDMT – The median time taken for a subject to release the response button and select the target stimulus after it flashed yellow on screen. Calculated across correct, assessed trials in which the stimulus could appear in one location only. Measured in milliseconds.<br>RTISMDRT – The median duration it took for a subject to release the response button after the presentation of a target stimulus. Calculated across correct, assessed trials in which the stimulus could appear in one location only. Measured in milliseconds. |
| **Rapid Visual Information Processing (RVP)** |
| **Domain:** sustained attention.<br>**Description:** participants have to press a button when they detect a target number sequence within a box of changing digits.<br>**Variables:**<br>RVPA – A′ (A prime) is the signal detection measure of a subject's sensitivity to the target sequence (string of three numbers), regardless of response tendency (the expected range is 0.00 to 1.00; bad to good). In essence, this metric is a measure of how good the subject is at detecting target sequences.<br>RVPMDL – The median response latency on trials where the subject responded correctly. Calculated across all assessed trials.<br>RVPPFA – The number of sequence presentations that were false alarms divided by the number of sequence presentations that were false alarms plus the number of sequence presentations that were correct rejections (False Alarms ÷ (False Alarms + Correct Rejections)) |
| **Stockings of Cambridge (SOC)** |
| **Domains:** spatial planning, working memory.<br>**Description:** participants move virtual balls to solve a spatial problem.<br>**Variables:**<br>SOCITMD5 – Subjects are encouraged to plan their moves before starting to solve the problems. Initial thinking time is the difference in the time taken to select the first ball for the same problem in the solve compared to follow conditions. This measure provides an indication of the time taken to plan the problem |

| |
|---|
| solution for assessed problems with 5 moves, discounting movement time. This score may be 0 if the subject is slower in the follow condition.<br>SOCMNM5 – The mean number of moves that the subject required to complete problems (calculated over 5 Moves assessed problems only)<br>SOCPSMMT – The number of assessed problems that the subject successfully completed in the minimum possible number of moves. Calculated over all assessed trials.<br>SOCSTMD5 – Calculated as the time between selecting the first ball and completing the problem in the solve conditions minus the corresponding time in the follow condition. This time difference is then divided by the number of moves made in the solve phase. This measure provides an indication of any time taken by the subject to plan or re-plan the problem solution after they have made their first move taking into account their movement time, and the number of moves made. Calculated across all 5 move problems. This score may be 0 if the subject is slower in the follow condition. |
| **Spatial Span (SSP)** |
| **Domain:** working memory capacity.<br>**Description:** participants must recall a sequence of boxes changing colour.<br>**Variable:**<br>SSPFSL – The longest sequence of boxes successfully recalled by the subject. |
| **Spatial Working Memory (SWM)** |
| **Domains:** working memory, strategy use.<br>**Description:** participants must find hidden tokens across a set of coloured boxes on the screen.<br>**Variables:**<br>SWMBE4 – The number of times a subject revisits a box in which a token has previously been found. Calculated across all trials with 4 tokens only.<br>SWMBE468 – The number of times the subject incorrectly revisits a box in which a token has previously been found. Calculated across all assessed four, six and eight token trials.<br>SWMBE6, SWMBE8 – The number of times the subject revisits a box in which a token has previously been found. Calculated across all trials with 6 (SWMBE6) or 8 (SWMBE8) tokens only.<br>SWMS – The number of times a subject begins a new search pattern from the same box they started with previously. If they always begin a search from the same starting point, it is inferred that the subject is employing a planned strategy for finding the tokens (a low score indicates high strategy use (1 = they always begin the search from the same box), a high score indicates that they are beginning their searches from many different boxes). Calculated across assessed trials with 6 tokens or more. |
